# Spontaneous passage of common bile duct stones: predictive factors and impact on post-ERCP complications

**DOI:** 10.1371/journal.pone.0351242

**Published:** 2026-07-02

**Authors:** Wasuwit Wanchaitanawong, Phichayut Phinyo, Thanaput Kunlayawutipong, Nawapol Chatkul, Phuripong Kijdamrongthum, Nithi Thinrungroj

**Affiliations:** 1 Division of Gastroenterology, Department of Internal Medicine, Faculty of Medicine, Chiang Mai University, Chiang Mai, Thailand; 2 Department of Biomedical Informatics and Clinical Epidemiology (BioCE), Faculty of Medicine, Chiang Mai University, Chiang Mai, Thailand; 3 Center for Clinical Epidemiology and Clinical Statistics, Faculty of Medicine, Chiang Mai University, Chiang Mai, Thailand; Universitatsklinikum Leipzig, GERMANY

## Abstract

**Background:**

Spontaneous passage of common bile duct stones (CBDSs) may render endoscopic retrograde cholangiopancreatography (ERCP) unnecessary. Although predictors of passage have been described, most prior studies were limited by a small number of events, and the impact of spontaneous passage on post-ERCP complications remains under-investigated. This study aimed to identify clinical predictors of spontaneous passage and evaluate its association with post-ERCP complications.

**Methods:**

We conducted a retrospective cohort study of patients diagnosed with CBDSs who underwent endoscopic ultrasonography (EUS) or ERCP at a tertiary referral center. Spontaneous passage was defined as the absence of stones confirmed during the procedure. Multivariable risk regression was used to identify predictors of passage and to assess the association between spontaneous passage and post-ERCP complications.

**Results:**

Spontaneous passage was observed in 113 of 404 patients (28%). Independent predictors of spontaneous passage included younger age (RR 0.88 per 10 years; 95% CI 0.81–0.96), smaller CBDS size (RR 0.78 per 1 mm; 95% CI 0.71–0.85), and single CBDS (RR 1.64; 95% CI 1.04–2.61). Regarding complications, post-ERCP pancreatitis (PEP) occurred more frequently in patients with spontaneous passage compared to those without (16.5% vs 7.6%, P = 0.01). After adjusting for relevant confounders, including procedural factors, spontaneous passage remained an independent risk factor for PEP (RR 2.48, 95% CI 1.25–4.92).

**Conclusions:**

Spontaneous passage of CBDSs is an independent risk factor for PEP. Younger age, smaller stone size, and a single stone are significant predictors of passage. These findings suggest that pre-procedural risk stratification and non-invasive confirmation of ductal clearance may be beneficial in selecting appropriate candidates, potentially reducing unnecessary ERCP and associated complications.

## Introduction

Common bile duct stones (CBDSs) are present in approximately 10–20% of patients with symptomatic gallstones [1]. If left untreated, CBDSs can lead to complications in up to 25% of cases within four years, including acute pancreatitis, acute cholangitis, and biliary tract obstruction [2]. Accordingly, international guidelines recommend endoscopic retrograde cholangiopancreatography (ERCP) with stone extraction for all patients with CBDSs [1, 3, 4]. However, spontaneous passage of CBDSs through the papilla occurs in 6–27% of patients, indicating that some may undergo unnecessary ERCP [5–15]. Smaller stone size is a well-established predictor of spontaneous passage [5,8,9,11,12,14,15]. Nevertheless, findings regarding other potential predictors remain inconsistent, and most studies have been limited by small numbers of spontaneous passage events.

ERCP is an invasive procedure associated with a 6–15% risk of complications, including post-ERCP pancreatitis (PEP), bleeding, infection, perforation, and sedation-related adverse events, which can be potentially fatal [16,17]. Whether spontaneous passage of CBDSs influences the risk of post-ERCP complications has not been well studied.

To address these gaps, this study aimed to (1) identify clinical predictors of spontaneous passage of CBDSs and (2) evaluate the association between spontaneous passage of CBDSs and post-ERCP complications.

## Materials and Methods

### Study design

This retrospective observational cohort study was conducted at Maharaj Nakorn Chiang Mai Hospital, a tertiary-care training center in Chiang Mai, Thailand. The study protocol was approved by the Research Ethics Committee of the Faculty of Medicine, Chiang Mai University, Thailand [MED-2565–09074]. Electronic medical records were accessed for research purposes between 3 August 2022 and 31 July 2023. The authors had access to identifiable information during the data extraction process. After data collection was completed, all datasets were anonymized prior to analysis to ensure participant confidentiality.

### Participants

We included all patients aged ≥15 years diagnosed with CBDSs by ultrasonography, computed tomography (CT), magnetic resonance imaging, magnetic resonance cholangiopancreatography (MRCP), or endoscopic ultrasonography (EUS), and who underwent ERCP or EUS that could confirm the presence of CBDSs between 1 January 2019 and 31 July 2022. The choice between ERCP and EUS was at the discretion of the attending endoscopist, based on clinical judgment and the perceived likelihood of spontaneous CBDSs passage. In selected patients, EUS was performed prior to ERCP despite prior imaging confirmation of CBDSs when there was a high clinical suspicion of spontaneous stone passage, to reassess ductal clearance and avoid unnecessary ERCP if stones were no longer present. Exclusion criteria were history of prior ERCP, prior biliary surgery or intervention, biliary stricture, biliary or periampullary neoplasm, severe cholangitis requiring urgent ERCP, and failed biliary cannulation or unsuccessful EUS that precluded confirmation of CBDSs.

### ERCP procedure

In this study, ERCP procedures were performed by experienced endoscopists or advanced endoscopy fellows under direct, hands-on expert supervision, adhering to a standardized institutional protocol at a tertiary academic center. Procedures were performed in the prone position using a side-view duodenoscope with appropriate sedation. After biliary cannulation, cholangiography was performed to identify CBDSs. Endoscopic sphincterotomy and/or papillary balloon dilatation were then performed as indicated. A balloon catheter, basket catheter, or mechanical lithotriptor was used for stone extraction. Stone extraction using a balloon catheter, with or without a basket catheter, was also attempted even when cholangiography did not reveal CBDSs to ensure that small stones not visible on the cholangiogram were not missed.

### EUS Procedure

EUS procedures were performed by experienced endoscopists or advanced endoscopy fellows under direct, hands-on expert supervision, using a linear EUS scope with moderate sedation. CBDSs were evaluated from three standard stations: the gastric station, the duodenal bulb, and the second portion of the duodenum.

### Data collection

Demographic data (age, sex, and presence of gallbladder), clinical presentation (symptomatic presentation, acute cholangitis, or acute pancreatitis), laboratory data [at initial presentation: alkaline phosphatase (ALP), aspartate aminotransferase (AST), alanine aminotransferase (ALT), and total bilirubin; pre-procedure: ALP and total bilirubin], imaging characteristics (maximal CBD diameter, CBDS size, CBDS location, and number of CBDSs), duration from diagnosis to procedure, procedure outcomes, and post-ERCP complications were collected through electronic chart review. Pre-procedure laboratory data were obtained within 48 hours prior to the procedure. Normalization of ALP was defined as ALP ≤ 129 U/L, which represents the upper limit of normal in our laboratory. Normalization of total bilirubin was defined as total bilirubin ≤ 1.5 mg/dL.

Spontaneous passage of CBDSs was defined as the absence of CBDSs or sludge retrieved during ERCP, or no CBDSs detected on EUS. For patients who underwent EUS with CBDSs still present and subsequently proceeded to ERCP, the final ERCP results were used to determine the outcome. Non-passage of CBDSs was defined as the presence of CBDSs or sludge retrieved during ERCP. Post-ERCP complications, including PEP, were defined according to the European Society of Gastrointestinal Endoscopy (ESGE) guideline [18]. PEP was defined as new-onset or worsening abdominal pain with a greater than threefold elevation of serum amylase or lipase measured at least 24 hours after ERCP, requiring hospital admission or prolongation of hospitalization. The severity of PEP was classified according to the revised Atlanta classification [19].

### Statistical analysis

All statistical analyses were performed using Stata version 18.5 (StataCorp, College Station, TX, USA). Categorical variables were summarized as frequencies and percentages, whereas continuous variables were expressed as mean ± standard deviation (SD) or median with range or interquartile range (IQR).

Univariable log-binomial regression was performed to assess the association between each variable and spontaneous passage of CBDSs. Given the exploratory nature of this study, multivariable analysis was conducted without variable selection; however, variables demonstrating high collinearity with others were excluded before multivariable modeling. Sensitivity analyses were performed restricting the cohort to patients in whom CBDSs were confirmed by ERCP only and who did not undergo EUS prior to ERCP.

Post-ERCP complications between patients with and without spontaneous passage of CBDSs were compared using Pearson’s chi-squared test or Fisher’s exact test, as appropriate. Complications showing statistically significant differences were further analyzed using multivariable models adjusted for confounding factors to evaluate the association between spontaneous passage of CBDSs and those complications. Confounder selection was guided by a directed acyclic graph [20].

Multivariable analysis was initially attempted using log-binomial regression. Due to non-convergence, a robust (modified) Poisson regression model was applied as an alternative, as it yields consistent point estimates for risk ratios [21]. A two-sided p-value < 0.05 was considered statistically significant.

## Results

**Fig 1:**
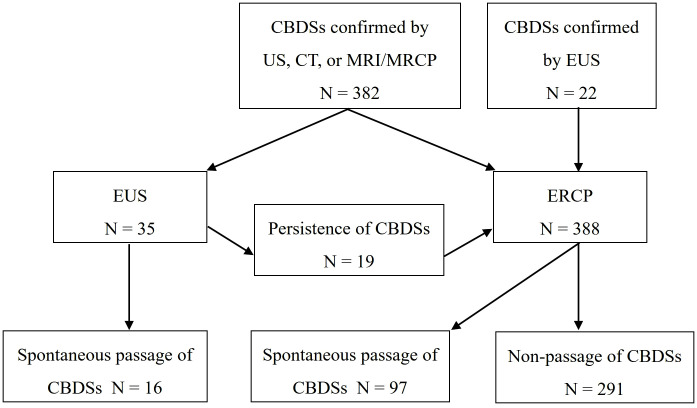
Study flow diagram. Abbreviation: US, ultrasonography; CT, Computed tomography; MRI, Magnetic resonance imaging; MRCP, magnetic resonance cholangiopancreatography; EUS, endoscopic ultrasonography; CBDS, common bile duct stone; ERCP, endoscopic retrograde cholangiopancreatography.

A total of 404 patients diagnosed with CBDSs were included in this study. Spontaneous passage occurred in 113 patients (28%), confirmed by EUS in 16 patients and by ERCP in 97 patients (Fig 1). Among the 388 patients who underwent ERCP, the stone clearance rate was 93.8%. Of the study population, 50% were male. The mean age was 63 years, and 93% were symptomatic. The mean duration from diagnosis to procedure was 29 days. Approximately 80% of patients were diagnosed with CBDSs by CT (Table 1).

**Table 1:** Baseline characteristics.

Characteristics	N = 404
Sex	
Male, n (%)	203 (50.2)
Female, n (%)	201 (49.8)
Age (years), mean (SD)	63 (16.5)
Clinical presentation	
Asymptomatic, n (%)	28 (6.9)
Symptomatic, n (%)	376 (93.1)
Abdominal pain, n (%)	369 (91.3)
Acute cholangitis, n (%)	249 (61.6)
Acute pancreatitis, n (%)	57 (14.1)
Gallbladder status	
Intact gallbladder, n (%)	342 (84.6)
Post cholecystectomy, n (%)	62 (15.4)
Duration from diagnosis to procedure (days), mean (SD, range)	28.6 (31.2, 0-287)
Within 7 days, n (%)	101 (25.0)
8-14 days, n (%)	65 (16.1)
15-21 days, n (%)	43 (10.7)
22-28 days, n (%)	51 (12.6)
More than 28 days, n (%)	144 (35.6)
Diagnostic modality	
Ultrasonography, n (%)	38 (9.4)
Computed tomography, n (%)	320 (79.2)
MRI/MRCP, n (%)	24 (6.0)
EUS, n (%)	22 (5.4)

Abbreviations: MRI, Magnetic resonance imaging; MRCP, magnetic resonance cholangiopancreatography; EUS, endoscopic ultrasonography; CBDS, common bile duct stone; ERCP, endoscopic retrograde cholangiopancreatography.

### Predictive factors for spontaneous passage of CBDSs

In univariable analysis, younger age, presentation with acute pancreatitis, smaller CBD diameter, smaller CBDS size, single CBDS, distal CBDS location, lower ALP, higher AST, and higher ALT were associated with spontaneous passage of CBDSs. Lower pre-procedure ALP and normalization of pre-procedure ALP were also associated with spontaneous passage (Table 2). These findings remained consistent in a sensitivity analysis restricted to patients in whom the presence of CBDSs was confirmed by ERCP only and who did not undergo EUS prior to ERCP (S1 Table).

**Table 2:** Univariable analysis of factors associated with spontaneous passage of common bile duct stones.

Factors	Spontaneous passage of CBDSs (n = 113)	Non-passage of CBDSs (n = 291)	Risk ratio (95% CI)	P value
**Demographic characteristics**				
Female, n (%)	60 (53.1)	141 (48.5)	1.14 (0.84-1.56)	0.40
Age (years), mean (SD)	54.7 (16.2)	66.8 (15.3)	0.77 (0.72-0.83)^†^	<0.01
Intact gallbladder, n (%)	97 (85.8)	245 (84.2)	1.10 (0.70-1.73)	0.68
**Clinical presentation**				
Symptomatic, n (%)	108 (95.6)	268 (92.1)	1.61 (0.72-3.62)	0.22
Acute cholangitis, n (%)	66 (58.4)	183 (62.9)	0.87 (0.64-1.20)	0.41
Acute pancreatitis, n (%)	24 (21.2)	33 (11.3)	1.64 (1.15-2.34)	0.01
**Imaging characteristics**				
CBD diameter (mm), mean (SD)	9.5 (3.9)	13.2 (5.4)	0.87 (0.84-0.91)	<0.01
CBDS size (mm), mean (SD)	4.9 (2.1)	9.2 (4.0)	0.72 (0.67-0.77)	<0.01
Single CBDS, n (%)	95 (84.1)	169 (58.1)	2.80 (1.77-4.43)	<0.01
Distal CBDS, n (%)	106 (95.5)	214 (75.6)	4.90 (2.07-11.59)	<0.01
**Initial laboratory parameters**				
ALP (U/L), median (IQR)	189.5 (135-292)	231.5 (142-384)	0.89 (0.80-0.98)^‡^	0.02
AST (U/L), median (IQR)	249.5 (102-458)	167.0 (66-313)	1.04 (1.02-1.06)^‡^	<0.01
ALT (U/L), median (IQR)	224.5 (97-466.5)	152.5 (59-274)	1.11 (1.07-1.15)^‡^	<0.01
TB (mg/dL), median (IQR)	2.6 (1.6-5.1)	2.5 (1.6-4.4)	1.01 (0.98-1.05)	0.53
**Pre-procedure parameters**				
Duration from diagnosis to procedure (days), mean (SD)	27.5 (23.5)	29.0 (33.7)	1.00 (0.99-1.00)	0.69
ALP (U/L), median (IQR)	107 (85-154)	147 (96-293)	0.77 (0.66-0.90)^‡^	<0.01
Normalization of ALP, n (%)	69 (65.7)	113 (43.0)	1.96 (1.38-2.77)	<0.01
TB (mg/dL), median (IQR)	0.8 (0.5-1.7)	0.8 (0.5-1.8)	0.99 (0.94-1.04)	0.62
Normalization of TB, n (%)	80 (74.8)	197 (72.7)	1.08 (0.74-1.57)	0.68

† Risk ratio is presented per 10-year increase in age. ‡ Risk ratios are presented per 100-U/L increase. Abbreviations: CBDS, common bile duct stone; CBD, common bile duct; ALP, alkaline phosphatase; AST, aspartate aminotransferase; ALT, alanine aminotransferase; TB, total bilirubin.

The overall duration from diagnosis to procedures did not differ between the two groups. Subsequently, we performed an exploratory analysis to evaluate the risk of spontaneous CBDS passage across different time intervals, using patients who underwent ERCP within 7 days as the reference group. Among those who underwent ERCP within the first 28 days, the risk of spontaneous passage increased progressively with longer intervals, with the highest and statistically significant risk observed in patients who underwent ERCP 22–28 days after diagnosis (RR 1.89, 95% CI 1.13–3.15), as shown in S2 Table.

We also evaluated the potential impact of diagnostic modality on spontaneous passage and found that there was no significant difference in the prevalence of spontaneous passage of CBDSs across modalities (p = 0.74), and no significant association between diagnostic modality and spontaneous passage (Table S3).

In multivariable analysis, younger age, smaller CBDS size, and single CBDS remained significantly associated with spontaneous passage of CBDSs (Table 3). These associations remained consistent in a sensitivity analysis restricted to patients in whom the presence of CBDSs was confirmed by ERCP only and who did not undergo EUS prior to ERCP (S4 Table).

**Table 3:** Multivariable analysis of factors associated with spontaneous passage of common bile duct stones.

Factors	Risk ratio	95% CI	P value
Female	1.25	0.93-1.66	0.13
Age	0.88^†^	0.81-0.96	<0.01
Intact gallbladder	0.82	0.55-1.23	0.34
Symptomatic presentation	1.78	0.70-4.56	0.23
Acute cholangitis	1.19	0.86-1.65	0.30
Acute pancreatitis	1.28	0.87-1.87	0.21
CBD diameter	0.98	0.92-1.04	0.47
CBDS size	0.78	0.71-0.85	<0.01
Single CBDS	1.64	1.04-2.61	0.03
Distal CBDS	1.63	0.79-3.37	0.19
ALP	1.04^‡^	0.95-1.14	0.44
ALT	1.03^‡^	0.98-1.09	0.20
TB	0.99	0.95-1.04	0.77
Duration from diagnosis to procedure	1.00	0.99-1.01	0.98
ALP normalization	1.24	0.86-1.80	0.25
TB normalization	1.05	0.67-1.62	0.84

† Risk ratio is presented per 10-year increase in age. ‡ Risk ratios are presented per 100-U/L increase. Abbreviations: CBD, common bile duct; CBDS, common bile duct stone; ALP, alkaline phosphatase; ALT, alanine aminotransferase; TB, total bilirubin.

### Association between spontaneous passage of CBDSs and post-ERCP complications

Overall post-ERCP complications tended to occur more frequently in patients with spontaneous passage of CBDSs compared with those without spontaneous passage (21.6% vs 13.4%, p = 0.052). PEP was significantly more common in patients with spontaneous passage than in those without (16.5% vs 7.6%, p = 0.01) (Table 4). However, the severity of PEP did not differ significantly between the two groups (mild, moderate, and severe = 87.5%, 12.5%, and 0% in the spontaneous passage group vs 86.4%, 9.1%, and 4.5% in the non-passage group; p = 0.66).

**Table 4:** Post-ERCP complications in patients with spontaneous passage of common bile duct stones compared with non-passage group.

Complications	Total (n = 388)	Spontaneous passage of CBDSs (n = 97)	Non-passage of CBDSs (n = 291)	P value
Overall, n (%)	60 (15.5)	21 (21.6)	39 (13.4)	0.052
Procedure-related complication, n (%)				
Pancreatitis	38 (9.8)	16 (16.5)	22 (7.6)	0.01
Bleeding	3 (0.8)	2 (2.1)	1 (0.3)	0.16^†^
Infection	21 (5.4)	4 (4.1)	17 (5.8)	0.61^†^
Perforation	2 (0.5)	0	2 (0.7)	>0.99^†^
Sedation-related complication, n (%)	3 (0.8)	1 (1.0)	2 (0.7)	>0.99^†^
Death, n (%)	1 (0.3)	0	1 (0.3)	>0.99^†^

† Fisher’s exact test. Abbreviations: CBDS, common bile duct stone.

**Fig 2:**
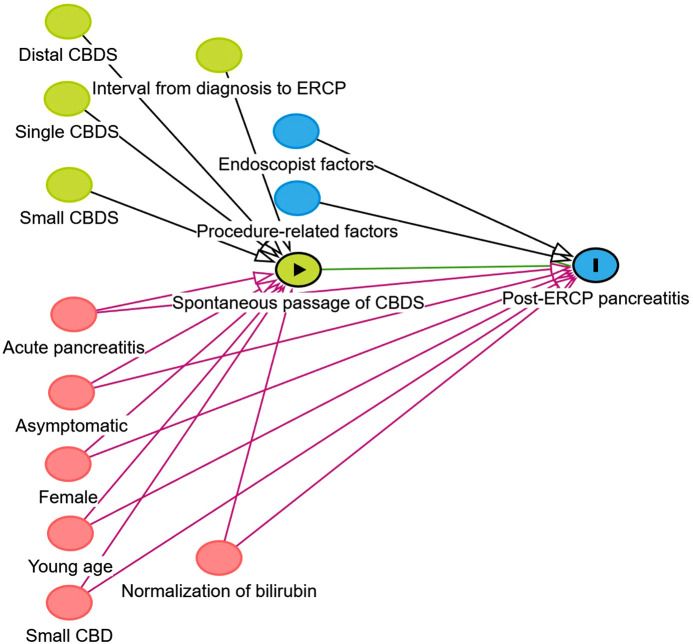
Directed acyclic graph illustrating the causal pathways between spontaneous passage of common bile duct stones and post-ERCP pancreatitis. Factors considered confounders are variables with arrows directed toward both spontaneous passage of CBDSs and post-ERCP pancreatitis. Abbreviations: ERCP, endoscopic retrograde cholangiopancreatography; CBDS, common bile duct stone; CBD, common bile duct.

A multivariable analysis was performed to further evaluate the association between spontaneous passage of CBDSs and PEP. Confounding factors for adjustment were selected based on a directed acyclic graph constructed from our study findings and literature review [18,22]. The covariates included female sex, younger age, prior acute pancreatitis, asymptomatic presentation, CBD diameter, and normalization of bilirubin (Fig 2). After adjustment for these factors, spontaneous passage of CBDSs remained an independent factor associated with PEP, with a risk ratio of 2.34 (95% CI, 1.15–4.75).

We further compared procedure-related factors between patients with and without PEP and found that pancreatic duct cannulation occurred more frequently among those who developed PEP (18.4% vs 3.4%, p < 0.01) (S5 Table). No pancreatic duct contrast injection was performed, and no rectal nonsteroidal anti-inflammatory drugs were used in this cohort. When procedural factors—including pancreatic duct cannulation, pancreatic duct stent placement, and precut sphincterotomy—were added to the previous multivariable model, spontaneous passage of CBDSs remained independently associated with PEP (RR 2.48, 95% CI 1.25–4.92).

## Discussion

In this retrospective cohort study, which included a relatively large number of patients with spontaneous passage of CBDSs (113 patients, 28%), the prevalence was slightly higher than that reported in previous studies, which ranged from 6% to 27% [5–15], and also higher than the pooled prevalence of 15.7% (95% CI 11.5%–21.2%) reported in a recent meta-analysis [23]. The higher prevalence likely reflects the longer mean duration from diagnosis of CBDSs to procedures (29 days), compared with 4–24 days in prior studies [7,9,11,12,14,15], which could allow more time for CBDSs to pass through the papilla spontaneously. The relatively prolonged interval in our cohort reflects both referral patterns to our tertiary center—where patients are often transferred after stabilization of the initial acute phase —and limited ERCP and EUS capacity, as these procedures are available in only a few centers in our region, thereby contributing to longer waiting times. This unintentional delay provided a unique window to observe the natural history of stone passage.

We identified several predictors associated with spontaneous passage of CBDSs. Smaller CBDS size was confirmed as a strong predictor of spontaneous passage, consistent with prior reports [5,8,9,11,12,14,15]. A recent meta-analysis similarly demonstrated this association, reporting a mean CBDS size of 3.8 mm among patients with spontaneous passage [23]. In our cohort, the mean CBDS size in the spontaneous passage group was slightly larger than that reported in the meta-analysis (4.9 mm). The appropriate cut-off for CBDS size used to predict spontaneous passage has varied across studies. Previous investigations have proposed thresholds ranging from 2 to 8 mm, each with differing performance [5–9,11,12,14,15]. Our study provides additional insight by demonstrating that each 1-mm increase in CBDS size reduces the likelihood of spontaneous passage, with a risk ratio of 0.78. This continuous effect may be incorporated into future clinical prediction models for more precise risk estimation. We also found that having a single CBDS was associated with spontaneous passage, consistent with previous reports [8,12,23]. Another imaging characteristic identified in prior studies is CBD diameter, which tends to be smaller in patients with spontaneous passage [12,23]. This was observed in our univariable analysis; however, the association was not retained in the multivariable model.

Previous studies have reported that patients with spontaneous passage tend to be younger [13,23], and our multivariable analysis confirmed this association. Increasing age was significantly associated with a lower likelihood of spontaneous passage, with the risk decreasing by an RR of 0.88 for every 10-year increase. The underlying mechanism of this association remains unclear and requires further investigation. Another demographic factor previously studied is female sex, which was reported in one study to be associated with a lower likelihood of spontaneous passage of CBDSs[11]. However, our findings, consistent with the meta-analysis, showed that sex was not a significant predictor [23].

Presentation with acute pancreatitis was not identified as a significant predictor in a recent meta-analysis (RR 1.60, 95% CI 0.81–3.17) [23]. However, a multicenter retrospective study reported that acute pancreatitis was a significant predictor of spontaneous passage in multivariable analysis (OR 2.78, 95% CI 1.14–6.73) [11]. In our study, univariable analysis similarly showed that acute pancreatitis increased the likelihood of spontaneous passage (RR 1.64, 95% CI 1.15–2.34). Nonetheless, in our more conservative multivariable model that adjusted for all potential predictive factors, acute pancreatitis was not a significant predictor (RR 1.28, 95% CI 0.87–1.87). For another clinical manifestation, acute cholangitis, our finding was consistent with the meta-analysis, which showed that acute cholangitis was not a significant predictor of spontaneous passage [23].

Two studies have reported that a longer interval from diagnosis to ERCP is associated with a higher likelihood of spontaneous passage of CBDSs in multivariable analysis [11,12], and this association was also consistent with a recent meta-analysis [23]. In contrast, our study did not observe this relationship in either univariable or multivariable analysis. However, our exploratory analysis demonstrated that, among patients who underwent ERCP within 28 days of diagnosis, the risk of spontaneous CBDSs passage increased progressively with longer intervals, with the highest and statistically significant risk observed in those who underwent ERCP 22–28 days after diagnosis. This may explain why prior studies—with mean procedure intervals of 5–6 days—identified duration as a significant predictor [11,12], whereas our study, which had a substantially longer mean interval of 29 days, did not show this association in the overall analysis.

Most studies have focused on predictive factors present at the time of CBDSs diagnosis; however, the likelihood of spontaneous passage of CBDSs may change over time, particularly when the interval between diagnosis and the procedure is prolonged. Because several parameters can change dynamically, it is also important to explore potential predictors measured closer to the procedure. A retrospective study from another center in Thailand demonstrated that resolution of clinical symptoms (absence of fever and abdominal pain) in patients with CBDSs and cholangitis predicted spontaneous passage [8]. A study from Portugal found that lower pre-ERCP total bilirubin increased the likelihood of spontaneous passage and proposed a cut-off of ≤ 2 mg/dL as a clinical decision guide [15]. In contrast, our study showed that pre-procedure bilirubin was not a significant predictor in either univariable or multivariable analyses. This discrepancy may be explained by the substantially longer mean interval between diagnosis and procedures in our cohort. Given the natural course of acute biliary obstruction, AST, ALT, and bilirubin levels typically decline spontaneously within a few days [24]; thus, in our cohort—with a relatively prolonged interval—the median bilirubin levels were already within the normal range and did not differ between groups. Furthermore, caution should be taken when using pre-procedure bilirubin as a predictor, as bilirubin levels may be affected in patients with bilirubin metabolism disorders or inherited hemoglobinopathies such as thalassemia, in which gallstone-related diseases are relatively common [25]. Our study demonstrated that lower pre-procedure ALP was associated with spontaneous passage in univariable analysis, potentially reflecting the slower decline of ALP after biliary obstruction. However, this association was not significant after multivariable adjustment.

Previous studies have reported no difference in post-ERCP complications between patients with spontaneous passage and those without; however, the number of complication cases in the spontaneous passage group was relatively small, which may have limited the statistical power to detect a difference [8,12,14,26]. A recent retrospective cohort study reported complication rates of 8% versus 4% in patients with and without spontaneous passage, respectively, although statistical comparison was not provided [15]. In our study, the overall complication rate also tended to be higher in patients with spontaneous passage, but the difference was not statistically significant. Nonetheless, the prevalence of PEP was significantly higher in the spontaneous passage group. Most cases were mild and the severity distribution did not differ between groups. We subsequently performed a multivariable analysis to evaluate the association between spontaneous passage of CBDSs and PEP. After adjusting for relevant confounders identified using a direct acyclic graph, spontaneous passage remained significantly associated with PEP. The association persisted after further adjustment for procedure-related factors. This association may be related to mechanisms similar to that proposed for acute biliary pancreatitis, in which transient ampullary obstruction from passing stones triggers pancreatic inflammation [27], followed by a second hit from mechanical manipulation during ERCP. Further studies are needed to clarify the mechanisms underlying this observation.

Our study highlights not only the issue of unnecessary ERCP but also the increased risk of PEP in patients with spontaneous passage of CBDSs. Developing and validating risk prediction tools that incorporate predictors identified in our study and others may help clinicians more accurately assess an individual patient’s likelihood of spontaneous passage and avoid the potential risks associated with ERCP. For patients with a high predicted likelihood of spontaneous passage, a highly accurate noninvasive test—such as EUS or MRCP—could be considered before proceeding to ERCP. These modalities show comparable diagnostic performance for detecting CBDSs, with EUS demonstrating a sensitivity of 95% and specificity of 97%, and MRCP demonstrating a sensitivity of 93% and specificity of 96% [28]. Supporting this, all 9 patients in our cohort who underwent EUS shortly before ERCP (on the same day or within one day) demonstrated persistent CBDSs on EUS, which were subsequently confirmed at ERCP. In clinical practice, EUS may offer a practical advantage, as it can be performed immediately before ERCP in the same procedural session, allowing real-time reassessment of ductal clearance and facilitating avoidance of unnecessary ERCP. In cases where noninvasive testing confirms the absence of stones, expectant management without ERCP is likely safe. Supporting this approach, a prospective study of patients with clinically suspected CBDSs but negative MRCP findings, followed for a mean of 10 months, reported only one case of recurrent CBDSs among 50 patients; this occurred in an individual with gallstones who had not undergone cholecystectomy [29]. However, in patients with CBDSs and acute cholangitis, the use of repeat noninvasive confirmation or expectant management—even when spontaneous passage is suspected—should be carefully weighed against potential risks and benefits, and further data on safety outcomes in this population are warranted. At present, ERCP remains the standard treatment recommended by international guidelines, particularly in patients with moderate to severe cholangitis requiring urgent intervention [30].

To our knowledge, this is the first study to demonstrate an increased risk of PEP in patients with spontaneous passage of CBDSs. Our cohort included a relatively large number of patients with spontaneous passage, and we applied robust statistical methods to evaluate both the predictors of spontaneous passage and its independent association with PEP. However, several limitations should be acknowledged. First, this was a retrospective study. Second, it was conducted at a single center, and the relatively long interval from diagnosis to procedures in our cohort may limit generalizability of the findings to other settings, particularly where procedures can be performed more rapidly. Third, CBDS confirmation was performed using heterogeneous methods—either EUS or ERCP. Nevertheless, EUS has high sensitivity and specificity for diagnosing CBDSs [28], making it highly comparable to ERCP. In addition, sensitivity analyses restricted to patients whose CBDSs were confirmed by ERCP alone yielded consistent results, supporting the robustness of our findings. Fourth, the diagnosis of CBDSs in our cohort relied on multiple imaging modalities. However, our exploratory analysis showed that diagnostic modality did not significantly influence the likelihood of spontaneous CBDSs passage. Another consideration is that each imaging modality carries a different potential false-positive rate, which may confound the observed prevalence of spontaneous CBDS passage. A study in which EUS was performed within 3 days prior to ERCP reported a false-positive rate of 1.6% (2 of 126 positive cases) [31]. Another study evaluating transabdominal ultrasonography and MRCP, performed within 4 hours to 2 weeks (mean 18 hours) before direct cholangiography, found that MRCP had a false-positive rate of 9.7% (3 of 31 cases), mainly due to misinterpretation of a prominent ampullary sphincter as a distal bile duct stone, whereas ultrasonography showed no false positive cases (0 of 13 cases) [32]. A study evaluating CT, with a mean interval of 7 days from CT to ERCP (range: same day to 15 days), reported false-positive rates ranging from 16.7% to 18.2% for non-contrast CT and from 27.8% to 35.0% for contrast-enhanced CT. These false-positive findings were attributed to opacified blood vessels adjacent to the bile duct or contrast enhancement of the bile duct mucosa [33]. However, because these studies included varied time intervals between imaging and ERCP, it is difficult to distinguish true false-positive findings from spontaneous stone passage. Notably, a study evaluating non-contrast CT performed within 1 hour prior to ERCP reported no false-positive cases among 15 patients with positive CT findings [34]. This suggests that the true false-positive rate of these imaging modalities may be lower than reported when diagnostic imaging is performed shortly before ERCP. Lastly, intraductal ultrasound or cholangioscopy was not performed during ERCP, so the possibility of missed small residual stones cannot be entirely excluded.

In conclusion, this study demonstrated that younger age, smaller CBDS size, and a single CBDS are significant predictors of spontaneous passage of CBDSs. Integrating these factors into clinical decision-making may help identify patients with a high likelihood of spontaneous passage who would benefit from noninvasive evaluation with EUS or MRCP to confirm the persistence of CBDSs. This approach has the potential to avoid ERCP in up to one-quarter of patients. Such a strategy would not only reduce unnecessary ERCP procedures but also decrease the risk of PEP, which we found to be associated with spontaneous passage of CBDSs. Future studies are warranted to develop and externally validate clinical prediction models for spontaneous passage of CBDSs, incorporating these factors to better guide individualized decision-making.

## Supporting information

S1 Table Univariable analysis of factors associated with spontaneous passage of common bile duct stones, restricted to patients with CBDSs diagnosed on initial imaging who underwent ERCP without prior EUS. (DOCX)

S2 Table Exploratory analysis of time intervals from diagnosis of CBDSs to procedure associated with spontaneous passage of common bile duct stones. (DOCX)

S3 Table Exploratory analysis of the association between diagnostic modality and spontaneous passage of common bile duct stones. (DOCX)

S4 Table Multivariable analysis of factors associated with spontaneous passage of common bile duct stones, restricted to patients with CBDSs diagnosed on initial imaging who underwent ERCP without prior EUS. (DOCX)

S5 Table Comparison of procedural factors between patients with and without post-ERCP pancreatitis. (DOCX)

S1 Data Anonymized raw data. (CSV)
