# Supplementary material for: Spontaneous passage of common bile duct stones: predictive factors and impact on post-ERCP complications
Source: PLoS One. 2026 Jul 2;21(7):e0351242. doi: 10.1371/journal.pone.0351242 (PMC13327282; doi:10.1371/journal.pone.0351242)
Supplement: S1 Table — (DOCX) [file pone.0351242.s001.docx]

**S1 Table**

| Factors | Spontaneous passage of CBDSs  (n = 85) | Non-passage of CBDSs  (n = 262) | Risk ratio  (95 % CI) | P value |
| --- | --- | --- | --- | --- |
| **Demographic** | | | | |
| Female, n (%) | 49 (57.6) | 127 (48.5) | 1.32 (0.91-1.92) | 0.14 |
| Age (years), mean (SD) | 54.5 (16.8) | 67.3 (15.2) | 0.75 (0.70-0.81)^†^ | <0.01 |
| Intact gallbladder, n (%) | 73 (85.9) | 220 (84.0) | 1.12 (0.66-1.92) | 0.67 |
| **Clinical presentation** | | | | |
| Symptomatic, n (%) | 82 (96.5) | 242 (92.4) | 1.94 (0.66-5.67) | 0.22 |
| Acute cholangitis, n (%) | 54 (63.5) | 170 (64.9) | 0.96 (0.65-1.40) | 0.82 |
| Acute pancreatitis, n (%) | 15 (17.7) | 25 (9.5) | 1.64 (1.05-2.58) | 0.04 |
| **Imaging characteristics** | | | | |
| CBD diameter (mm), mean (SD) | 9.9 (4.1) | 13.6 (5.3) | 0.87 (0.83-0.91) | <0.01 |
| CBDS size (mm), mean (SD) | 5.2 (2.2) | 9.5 (4.0) | 0.71 (0.66-0.77) | <0.01 |
| Single CBDS, n (%) | 74 (87.1) | 151 (57.6) | 3.65 (2.01-6.61) | <0.01 |
| Distal CBDS, n (%) | 81 (95.3) | 190 (74.5) | 5.2 (1.96-13.58) | <0.01 |
| **Initial laboratory parameters** | | | | |
| ALP (U/L), median (IQR) | 189 (135-279) | 232 (143-404) | 0.86 (0.77-0.98)^‡^ | 0.02 |
| AST (U/L), median (IQR) | 254 (102-454) | 169 (72-326) | 1.06 (1.00-1.11)^‡^ | 0.03 |
| ALT (U/L), median (IQR) | 225 (95-441) | 160 (67-293) | 1.14 (1.09-1.20)^‡^ | <0.01 |
| TB (mg/dL), median (IQR) | 2.9 (1.8-5.2) | 2.5 (1.7-4.5) | 1.02 (0.98-1.06) | 0.31 |
| **Pre-procedure parameters** | | | | |
| Duration from diagnosis to procedure (days), mean (SD) | 28.5 (25.5) | 29.0 (34.4) | 1.00 (0.99-1.00) | 0.91 |
| ALP (U/L), median (IQR) | 109 (85-156) | 152 (96-294) | 0.69 (0.56-0.85)^‡^ | <0.01 |
| Normalization of ALP, n (%) | 50 (63.3) | 100 (41.8) | 1.93 (1.29-2.88) | <0.01 |
| TB (mg/dL), median (IQR) | 0.8 (0.5-2.0) | 0.8 (0.5-1.8) | 1.00 (0.95-1.05) | >0.99 |
| Normalization of TB, n (%) | 57 (70.4) | 176 (72.1) | 0.94 (0.62-1.41) | 0.76 |

^†^Risk ratio is presented per 10-year increase in age. ^‡^Risk ratios are presented per 100-U/L increase. Abbreviations: CBDS, common bile duct stone; CBD, common bile duct; ALP, alkaline phosphatase; AST, aspartate aminotransferase; ALT, alanine aminotransferase; TB, total bilirubin.
