# Supplementary material for: Spontaneous passage of common bile duct stones: predictive factors and impact on post-ERCP complications
Source: PLoS One. 2026 Jul 2;21(7):e0351242. doi: 10.1371/journal.pone.0351242 (PMC13327282; doi:10.1371/journal.pone.0351242)
Supplement: S2 Table — (DOCX) [file pone.0351242.s002.docx]

**S2 Table**

| Factors | Risk ratio  (95 % CI) | P value |
| --- | --- | --- |
| Duration ≤7 days | reference | - |
| Duration 8-14 days | 1.48 (0.87-2.51) | 0.145 |
| Duration 15-21 days | 1.68 (0.96-2.93) | 0.069 |
| Duration 22-28 days | 1.89 (1.13-3.15) | 0.015 |
| Duration > 28 days | 1.24 (0.77-1.98) | 0.379 |
