# Supplementary material for: Spontaneous passage of common bile duct stones: predictive factors and impact on post-ERCP complications
Source: PLoS One. 2026 Jul 2;21(7):e0351242. doi: 10.1371/journal.pone.0351242 (PMC13327282; doi:10.1371/journal.pone.0351242)
Supplement: S3 Table — (DOCX) [file pone.0351242.s003.docx]

**S3 Table**

| Diagnostic modalities | Proportion of spontaneous passage of CBDSs | P value | Risk ratio (95% CI) | P value |
| --- | --- | --- | --- | --- |
| Ultrasonography | 26.3% | 0.74 | Reference group | - |
| Computed tomography | 27.2% |  | 1.03 (0.58-1.81) | 0.91 |
| MRI/MRCP | 33.3% |  | 1.27 (0.58-2.75) | 0.55 |
| EUS | 36.4% |  | 1.38 (0.64-2.98) | 0.41 |

Abbreviations: CBDS, common bile duct stone; EUS, endoscopic ultrasonography; MRI, Magnetic resonance imaging; MRCP, magnetic resonance cholangiopancreatography
