# Supplementary material for: Spontaneous passage of common bile duct stones: predictive factors and impact on post-ERCP complications
Source: PLoS One. 2026 Jul 2;21(7):e0351242. doi: 10.1371/journal.pone.0351242 (PMC13327282; doi:10.1371/journal.pone.0351242)
Supplement: S4 Table — (DOCX) [file pone.0351242.s004.docx]

**S4 Table**

| Factors | Risk ratio | 95% CI | P value |
| --- | --- | --- | --- |
| Female | 1.33 | 0.91-1.94 | 0.14 |
| Age | 0.89^†^ | 0.81-0.99 | 0.03 |
| Intact gallbladder | 0.69 | 0.43-1.10 | 0.12 |
| Symptomatic presentation | 2.42 | 0.83-7.03 | 0.11 |
| Acute cholangitis | 1.05 | 0.68-1.61 | 0.84 |
| Acute pancreatitis | 1.64 | 0.96-2.79 | 0.07 |
| CBD diameter | 0.96 | 0.89-1.05 | 0.39 |
| CBDS size | 0.78 | 0.70-0.86 | <0.01 |
| Single CBDS | 2.03 | 1.17-3.54 | 0.01 |
| Distal CBDS | 1.62 | 0.74-3.50 | 0.23 |
| ALP | 1.03^‡^ | 0.92-1.15 | 0.60 |
| ALT | 1.02^‡^ | 0.96-1.09 | 0.45 |
| TB | 1.00 | 0.95-1.06 | 0.94 |
| Duration from diagnosis to procedure | 1.00 | 1.00-1.01 | 0.22 |
| ALP normalization | 1.23 | 0.83-1.83 | 0.30 |
| TB normalization | 0.99 | 0.62-1.61 | 0.98 |

^†^Risk ratio is presented per 10-year increase in age. ^‡^Risk ratios are presented per 100-U/L increase. Abbreviations: ALP, alkaline phosphatase; ALT, alanine aminotransferase; CBD, common bile duct; CBDS, common bile duct stone; TB, total bilirubin.
