# Supplementary material for: Spontaneous passage of common bile duct stones: predictive factors and impact on post-ERCP complications
Source: PLoS One. 2026 Jul 2;21(7):e0351242. doi: 10.1371/journal.pone.0351242 (PMC13327282; doi:10.1371/journal.pone.0351242)
Supplement: S5 Table — (DOCX) [file pone.0351242.s005.docx]

**S5 Table**

| Procedure-related factors | No post-ERCP pancreatitis  (n = 350) | Post-ERCP pancreatitis  (n = 38) | P value |
| --- | --- | --- | --- |
| Pancreatic duct canulation, n (%) | 12 (3.4) | 7 (18.4) | <0.01 |
| Contrast injection into the pancreatic duct, n (%) | 0 | 0 | - |
| Pancreatic duct stent placement, n (%) | 9 (2.6) | 3 (7.9) | 0.10^†^ |
| Precut sphincterotomy, n (%) | 3 (0.9) | 0 | >0.99^†^ |

^†^Fisher’s exact test. Abbreviations: ERCP, endoscopic retrograde cholangiopancreatography.
