# Supplementary material for: Spontaneous passage of common bile duct stones: predictive factors and impact on post-ERCP complications
Source: PLoS One. 2026 Jul 2;21(7):e0351242. doi: 10.1371/journal.pone.0351242 (PMC13327282; doi:10.1371/journal.pone.0351242)
Supplement: Attachment — Submitted filename: Response to reviewers_4-5-2026.docx [file pone.0351242.s007.docx]

**Response to the Editors and Reviewers**

We sincerely thank the editors and reviewers for the thorough evaluation of our manuscript and for provide us the opportunity to revise our manuscript. We have revised the manuscript accordingly and provided a point-by-point response. All changes have been incorporated into the revised version of the manuscript with track changes. We hope that our responses and revisions would substantially improve the quality of our manuscript and would be qualified for publication in the PLOS ONE journal.

**Editor Comments:

Dear authors, thank you for your submission.

Your manuscript addresses a relevant clinical question. The manuscript was assessed by two external reviewers and myself. Although your findings are of interest, the main conclusion requires substantial revision before it can be considered further. In particular, the analysis of post-ERCP pancreatitis needs more adjustment for procedural confounders and key sensitivity analyses should be added.**

**Response:**

We sincerely thank the editor for the guidance. We have now included additional procedure-related factors in the model evaluating the association between spontaneous CBDS passage and post-ERCP pancreatitis, and the results remained robust. We also performed sensitivity analyses restricted to patients who underwent ERCP only without prior EUS, and the findings remained significant.

**Please pay attention on predictive and causal interpretations which should be more clearly separated.**

**Response:**

We appreciate the editor’s guidance. We have revised the manuscript to more clearly distinguish between predictive and causal interpretations.

**The recommendation of an “EUS-first” strategy should be discussed more conservatively and aligned with current guideline-based risk stratification.**

**Response:**

We thank the editor for this suggestion. We have revised the Discussion to present the applicability of our findings in a more conservative manner and to align it with current guideline recommendations.

**We therefore invite a thoroughly revised version that addresses these points and further issues raised by the two reviewers in depth.**

**Reviewers' comments:**

**Reviewer #1: The manuscript entitled “Spontaneous passage of common bile duct stones: predictive factors and impact on post-ERCP complications” aimed to identify clinical predictors of spontaneous passage and evaluate its association with post-ERCP complications. The authors concluded that spontaneous passage of CBDSs is an independent risk factor for PEP and younger age, smaller stone size, and a single stone are significant predictors of spontaneous passage.
The manuscript is well written, novel and the results may be of clinical relevance. However, there are some comments that need to be addressed.

- The diagnosis of CBDS was made using various imaging techniques. False-positive results are also possible. Please discuss the ‘false-positive rate’ for each technique and mention this in the text.**

**Response:**

We thank the reviewer for this suggestion. We agree that each imaging modality carries a different potential false-positive rate, which may confound the observed prevalence of spontaneous CBDS passage. A study in which EUS was performed within 3 days prior to ERCP reported a false-positive rate of 1.6% (2 of 126 positive cases) [1]. Another study evaluating transabdominal ultrasonography and MRCP, performed within 4 hours to 2 weeks (mean 18 hours) before direct cholangiography, found that MRCP had a false-positive rate of 9.7% (3 of 31 cases), mainly due to misinterpretation of a prominent ampullary sphincter as a distal bile duct stone, whereas ultrasonography showed no false positive cases (0 of 13 cases)[2]. A study evaluating CT, with a mean interval of 7 days from CT to ERCP (range: same day to 15 days), reported false-positive rates ranging from 16.7% to 18.2% for non-contrast CT and from 27.8% to 35.0% for contrast-enhanced CT. These false-positive findings were attributed to opacified blood vessels adjacent to the bile duct or contrast enhancement of the bile duct mucosa[3].

However, because these studies included varied time intervals between imaging and ERCP, it is difficult to distinguish true false-positive findings from spontaneous CBDS passage. Another study evaluating non-contrast CT performed within 1 hour prior to ERCP reported no false-positive cases among 15 patients with positive CT findings[4]. This suggests that the true false-positive rate of these imaging modalities may be lower than reported when diagnostic imaging is performed shortly before ERCP.

In addition, we performed an exploratory analysis showing that diagnostic modality was not significantly associated with spontaneous CBDS passage (S3 Table).

We have added these points to the limitations section of the Discussion.

**- The risk of PEP is influenced not only by patient-related factors but also, to a significant extent, by factors related to the examination. Factors such as cannulation of the pancreatic duct, contrast injection into the pancreatic duct and placement of a protective stent in the pancreatic duct were not mentioned. Nor was it mentioned whether rectal NSAIDs are administered for PEP prophylaxis. Please mention and discuss.**

**Response:**

We thank the reviewer for suggesting this important issue. We have now added S5 Table, which compares procedural factors between patients with and without post-ERCP pancreatitis. We have also included pancreatic duct cannulation, pancreatic duct stent placement, and pre-cut sphincterotomy as additional covariates in the model. This has strengthened the robustness of our findings, with spontaneous passage of CBDSs remaining associated with post-ERCP pancreatitis (risk ratio 2.48; 95% CI 1.25-4.92).

We did not adjust for pancreatic duct contrast injection, as this was not performed in our cohort, or for rectal NSAID use, as these were not available in our region. These additions and clarifications have been incorporated into the Results and in the Discussion sections.

**-What was the technical success rate of the ERCPs? How often was a pre-cut papillotomy necessary?**

**Response:**

In our eligibility criteria, cases with failed biliary cannulation were not included. Therefore, all included ERCP procedures achieved successful biliary access, as clarified in the Methods section. We have now reported the rate of complete stone clearance in the Results, which was achieved in 93.8% of cases. The frequency of pre-cut sphincterotomy was 0.8%, and these data have also been provided in S5 Table.

**-Even a spontaneous stone passage can lead to pancreatitis. How was the distinction made between PEP and biliary pancreatitis caused by a spontaneous stone passage if this occurred shortly before the ERCP? If a clear distinction cannot be made, this should be noted under ‘possible limitations’.**

**Response:**

We thank the reviewer for raising this important point. There were two patients in our cohort who initially presented with acute pancreatitis and subsequently developed PEP. The intervals between the diagnosis of pancreatitis and ERCP in these patients were 36 and 13 days, respectively, and both had complete resolution of symptoms prior to ERCP. Therefore, the PEP events observed in our study were clearly incident cases and were consistent with the definition of PEP according to ESGE guidelines, defined as new-onset or worsening abdominal pain with a greater than threefold elevation of serum amylase or lipase measured at least 24 hours after ERCP, requiring hospital admission or prolongation of hospitalization [5].

In addition, we have elaborated on the definition of PEP in the Methods section.

**- The mean duration from diagnosis of CBDS to procedure (ERCP/EUS) was 29 days. That is a quiet long time. This is surprising, given that a large proportion of patients presented with acute cholangitis (Table 1). National and international guidelines recommend early ERCP for acute cholangitis. This should be discussed, as should the limited applicability of these data to other patient groups who have easier access to prompt ERCP.**

**Response:**

We thank the reviewer for this suggestion. The relatively prolonged interval in our cohort reflects both referral patterns to our tertiary center—where patients are often transferred after stabilization of the initial acute phase—and limited ERCP and EUS capacity, as these procedures are available in only a few centers in our region, thereby contributing to longer waiting times.

We agree that this may limit the generalizability of our findings to other settings, particularly those where procedures can be performed more rapidly. We have now addressed this point in the limitations section.

**- Why was an EUS performed on some patients prior to a potential ERCP, despite their previously confirmed CBDS? Please explain.**

**Response:**

We thank the reviewer for this important question. The choice between ERCP and EUS was at the discretion of the attending endoscopist, based on clinical judgment and the perceived likelihood of spontaneous CBDS passage. In selected patients, EUS was performed prior to ERCP despite prior imaging confirmation of CBDSs when there was a high clinical suspicion of spontaneous stone passage, in order to reassess ductal clearance and avoid unnecessary ERCP if stones were no longer present. We have added this explanation to the Methods section.

**Reviewer #2: In this manuscript Wanchaitanawong et al. report on the frequency and predictors of spontaneous passage of common bile duct stones in a retrospective cohort of 113 patients. The observed spontaneous passage in 28% of the patients and identify several factors associated with this outcome. Most importantly, they also observe a higher number of complications in patients with spontaneous passage underscoring the need to identify these before the procedure.
The study is technically sound and the manuscript is well written. The data are interesting.
However, a few points need to be considered:

- While the various predictors of spontaneous passage are certainly interesting, one very important aspect is inadequately covered: The predictive value of confirmation or exclusion of CBDS by appropriate imaging DIRECTLY before the ERCP. To this end, it is important to include this into the list of predictors. Roughly estimated, the p-value for positive EUS to exclude spontaneous CBDS passage should be 0.005 in this cohort and is therefore certainly relevant.**

**Response:**

We thank the reviewer for this valuable suggestion. We agree that evaluating the predictive value of imaging performed directly before ERCP would be clinically informative. However, in our cohort, confirmatory procedures (ERCP or EUS) were only performed in patients with previously positive imaging for CBDS, as our objective was not to assess the diagnostic performance of each imaging modality. Therefore, we did not have data on patients with negative imaging findings to construct 2×2 tables or formally estimate the diagnostic performance of each imaging modality.

Nevertheless, we have added descriptive data regarding patients who underwent EUS shortly before ERCP in the Discussion section. Specifically, nine patients underwent EUS within one day prior to ERCP; all were positive for CBDS on EUS, and persistence of CBDS was subsequently confirmed by ERCP in all cases. This finding supports the potential usefulness of EUS performed immediately before ERCP to confirm stone persistence.

**- This is also the most important and clinically relevant conclusion of the manuscript and should be made clearer in the discussion: In particular in cases with a substantial delay between diagnosis and intervention, EUS should be performed directly prior to ERCP to confirm or rule out CBDS.**

**Response:**

We thank the reviewer for this important suggestion. As noted above, we were not able to formally estimate the predictive performance of EUS in our cohort due to the lack of patients with negative CBDS on EUS who subsequently underwent ERCP. Therefore, we are unable to draw a strong conclusion regarding the routine use of EUS directly prior to ERCP.

However, we have revised the Discussion to more clearly highlight the potential benefit of using EUS, based on the existing literature, findings from a subgroup of patients who underwent EUS shortly before ERCP, and considerations from real-world clinical setting.

**- To identify cases, where the cumulative evidence from clinical predictors for spontaneous stone passage is so high that re-EUS might be omitted, I suggest the authors build a predictive model from these factors and indicate its sensitivity, specificity, NPV and PPV.**

**Response:**

We appreciate the reviewer’s suggestion. We agree that developing a predictive model would add value in terms of clinical applicability. However, the present study was primarily designed to explore predictors of spontaneous CBDS passage and to evaluate its association with post-ERCP complications.

In addition, a key concern regarding model development in this dataset is the limited sample size. As suggested by Riley et al.[6], to develop a prediction model with an expected C-statistic ≥ 0.75, given a prevalence of spontaneous CBDS passage of 0.28 and approximately 10 candidate predictors (based on significant variables in univariable analysis, Table 2), a minimum total sample size of 531 patients and at least 149 spontaneous passage events would be required. The detailed calculation process is provided in the figure below.

Therefore, our current sample size may be insufficient for robust model development.

Therefore, our current sample size may be insufficient for robust model development. We plan to collect additional data to support future model development. We have also added this suggestion as a potential direction for future research in the Conclusion section.

**- The study design comes with the weakness that a subgroup of patients has previously undergone EUS. Thus, the passage group includes a subgroup that has not undergone ERCP and the non-passage group includes a subgroup, where non-passage has already been confirmed before ERCP. This introduces some - probably minor - inconsistencies. However, I suggest to provide (e.g. as Supplementary Data) a separate analysis only focusing on the ERCP cases without previous EUS.**

**Response:**

We thank the reviewer for this suggestion. We have now added sensitivity analyses (S1 and S4 Tables), as suggested. The results remained consistent, supporting the robustness of our findings.

**- Please analyze whether the type of diagnostic mortality is associated with stone passage. Given the high number of diagnoses made with CT scan, I suspect that a high number of CBDS in this group were incidental findings on imaging performed for another indication.**

**Response:**

We thank the reviewer for this insightful comment. In our cohort, 28 patients (6.9%) were asymptomatic, of whom 18 were diagnosed by CT. This proportion was relatively low, and we found that symptomatic status was not associated with spontaneous CBDS passage (Table 2).

We have now performed an analysis to evaluate the association between diagnostic modality and spontaneous CBDS passage. We found that the proportion of patients with spontaneous CBDS passage did not differ across diagnostic modalities (p = 0.74), and there was no significant association between diagnostic modality and spontaneous CBDS passage (S3 Table). These findings suggest that our results were not significantly influenced by differences in diagnostic modality.

**References**

1.	Wang M, He X, Tian C, Li J, Min F, Li HY. The Diagnostic Accuracy of Linear Endoscopic Ultrasound for Evaluating Symptoms Suggestive of Common Bile Duct Stones. Gastroenterol Res Pract. 2016;2016:6957235. Epub 20160817. doi: 10.1155/2016/6957235. PubMed PMID: 27610131; PubMed Central PMCID: PMCPMC5005564.

2.	Varghese JC, Liddell RP, Farrell MA, Murray FE, Osborne DH, Lee MJ. Diagnostic accuracy of magnetic resonance cholangiopancreatography and ultrasound compared with direct cholangiography in the detection of choledocholithiasis. Clin Radiol. 2000;55(1):25-35. doi: 10.1053/crad.1999.0319. PubMed PMID: 10650107.

3.	Anderson SW, Lucey BC, Varghese JC, Soto JA. Accuracy of MDCT in the diagnosis of choledocholithiasis. AJR Am J Roentgenol. 2006;187(1):174-80. doi: 10.2214/AJR.05.0459. PubMed PMID: 16794173.

4.	Jiménez Cuenca I, del Olmo Martínez L, Pérez Homs M. Helical CT without contrast in choledocholithiasis diagnosis. European Radiology. 2001;11(2):197-201. doi: 10.1007/s003300000609.

5.	Dumonceau JM, Kapral C, Aabakken L, Papanikolaou IS, Tringali A, Vanbiervliet G, et al. ERCP-related adverse events: European Society of Gastrointestinal Endoscopy (ESGE) Guideline. Endoscopy. 2020;52(2):127-49. Epub 20191220. doi: 10.1055/a-1075-4080. PubMed PMID: 31863440.

6.	Riley RD, Ensor J, Snell KIE, Harrell FE, Jr., Martin GP, Reitsma JB, et al. Calculating the sample size required for developing a clinical prediction model. BMJ. 2020;368:m441. Epub 20200318. doi: 10.1136/bmj.m441. PubMed PMID: 32188600.
