# Supplementary material for: Spontaneous passage of common bile duct stones: predictive factors and impact on post-ERCP complications
Source: PLoS One. 2026 Jul 2;21(7):e0351242. doi: 10.1371/journal.pone.0351242 (PMC13327282; doi:10.1371/journal.pone.0351242)
Supplement: Peer review history

# Peer review: Decision Letter 0

**DOI:** 10.1371/journal.pone.0351242.r001

**Authors:** Thomas Karlas

13 Apr 2026

PONE-D-26-05028

Spontaneous passage of common bile duct stones: predictive factors and impact on post-ERCP complications

PLOS One

Dear Dr. Thinrungroj,

Thank you for submitting your manuscript to PLOS ONE. After careful consideration, we feel that it has merit but does not fully meet PLOS ONE’s publication criteria as it currently stands. Therefore, we invite you to submit a revised version of the manuscript that addresses the points raised during the review process (see below).

Please submit your revised manuscript by May 28 2026 11:59PM. If you will need more time than this to complete your revisions, please reply to this message or contact the journal office at plosone@plos.org. When you're ready to submit your revision, log on to https://www.editorialmanager.com/pone/ and select the 'Submissions Needing Revision' folder to locate your manuscript file.

Please include the following items when submitting your revised manuscript:

- A letter that responds to each point raised by the academic editor and reviewer(s). You should upload this letter as a separate file labeled 'Response to Reviewers'.
- A marked-up copy of your manuscript that highlights changes made to the original version. You should upload this as a separate file labeled 'Revised Manuscript with Track Changes'.
- An unmarked version of your revised paper without tracked changes. You should upload this as a separate file labeled 'Manuscript'.

If you would like to make changes to your financial disclosure, please include your updated statement in your cover letter. Guidelines for resubmitting your figure files are available below the reviewer comments at the end of this letter.

If applicable, we recommend that you deposit your laboratory protocols in protocols.io to enhance the reproducibility of your results. Protocols.io assigns your protocol its own identifier (DOI) so that it can be cited independently in the future. For instructions see: https://journals.plos.org/plosone/s/submission-guidelines#loc-laboratory-protocols. Additionally, PLOS ONE offers an option for publishing peer-reviewed Lab Protocol articles, which describe protocols hosted on protocols.io. Read more information on sharing protocols at https://plos.org/protocols?utm_medium=editorial-email&utm_source=authorletters&utm_campaign=protocols.

As the corresponding author, your ORCID iD is verified in the submission system and will appear in the published article. PLOS supports the use of ORCID, and we encourage all coauthors to register for an ORCID iD and use it as well. Please encourage your coauthors to verify their ORCID iD within the submission system before final acceptance, as unverified ORCID iDs will not appear in the published article. *Only*  the individual author can complete the verification step; PLOS staff *cannot*  verify ORCID iDs on behalf of authors.

We look forward to receiving your revised manuscript.

Kind regards,

Thomas Karlas

Academic Editor

PLOS One

Journal Requirements:

When submitting your revision, we need you to address these additional requirements.

1. Please ensure that your manuscript meets PLOS ONE's style requirements, including those for file naming. The PLOS ONE style templates can be found at

https://journals.plos.org/plosone/s/file?id=wjVg/PLOSOne_formatting_sample_main_body.pdf and https://journals.plos.org/plosone/s/file?id=ba62/PLOSOne_formatting_sample_title_authors_affiliations.pdf

2. We note that you have indicated that there are restrictions to data sharing for this study. For studies involving human research participant data or other sensitive data, we encourage authors to share de-identified or anonymized data. However, when data cannot be publicly shared for ethical reasons, we allow authors to make their data sets available upon request. For information on unacceptable data access restrictions, please see http://journals.plos.org/plosone/s/data-availability#loc-unacceptable-data-access-restrictions.

Before we proceed with your manuscript, please address the following prompts:

a) If there are ethical or legal restrictions on sharing a de-identified data set, please explain them in detail (e.g., data contain potentially identifying or sensitive patient information, data are owned by a third-party organization, etc.) and who has imposed them (e.g., a Research Ethics Committee or Institutional Review Board, etc.). Please also provide contact information for a data access committee, ethics committee, or other institutional body to which data requests may be sent.

b) If there are no restrictions, please upload the minimal anonymized data set necessary to replicate your study findings to a stable, public repository and provide us with the relevant URLs, DOIs, or accession numbers. Please see http://www.bmj.com/content/340/bmj.c181.long for guidelines on how to de-identify and prepare clinical data for publication. For a list of recommended repositories, please see https://journals.plos.org/plosone/s/recommended-repositories. You also have the option of uploading the data as Supporting Information files, but we would recommend depositing data directly to a data repository if possible.

Please update your Data Availability statement in the submission form accordingly.

3. In the online submission form, you indicated that the data is not publicly available due to institutional policies and patient privacy regulations. However, de-identified data may be available from the corresponding author upon reasonable request and subject to institutional approval.

All PLOS journals now require all data underlying the findings described in their manuscript to be freely available to other researchers, either 1. In a public repository, 2. Within the manuscript itself, or 3. Uploaded as supplementary information.

This policy applies to all data except where public deposition would breach compliance with the protocol approved by your research ethics board. If your data cannot be made publicly available for ethical or legal reasons (e.g., public availability would compromise patient privacy), please explain your reasons on resubmission and your exemption request will be escalated for approval.

4. If the reviewer comments include a recommendation to cite specific previously published works, please review and evaluate these publications to determine whether they are relevant and should be cited. There is no requirement to cite these works unless the editor has indicated otherwise.

Additional Editor Comments :

Dear authors, thank you for your submission.

Your manuscript addresses a relevant clinical question. The manuscript was assessed by two external reviewers and myself. Although your findings are of interest, the main conclusion requires substantial revision before it can be considered further. In particular, the analysis of post-ERCP pancreatitis needs more adjustment for procedural confounders and key sensitivity analyses should be added. Please pay attention on predictive and causal interpretations which should be more clearly separated. The recommendation of an “EUS-first” strategy should be discussed more conservativelly and aligned with current guideline-based risk stratification.

We therefore invite a thoroughly revised version that addresses these points and further issues raised by the two reviewers in depth.

[Note: HTML markup is below. Please do not edit.]

Reviewers' comments:

Reviewer's Responses to Questions

**Comments to the Author**

1. Is the manuscript technically sound, and do the data support the conclusions?

The manuscript must describe a technically sound piece of scientific research with data that supports the conclusions. Experiments must have been conducted rigorously, with appropriate controls, replication, and sample sizes. The conclusions must be drawn appropriately based on the data presented.

Reviewer #1: Yes

Reviewer #2: Yes

**********

2. Has the statistical analysis been performed appropriately and rigorously?

Reviewer #1: Yes

Reviewer #2: Yes

**********

3. Have the authors made all data underlying the findings in their manuscript fully available?

The PLOS Data policy requires authors to make all data underlying the findings described in their manuscript fully available without restriction, with rare exception (please refer to the Data Availability Statement in the manuscript PDF file). The data should be provided as part of the manuscript or its supporting information, or deposited to a public repository. For example, in addition to summary statistics, the data points behind means, medians and variance measures should be available. If there are restrictions on publicly sharing data—e.g. participant privacy or use of data from a third party—those must be specified.

Reviewer #1: Yes

Reviewer #2: Yes

**********

4. Is the manuscript presented in an intelligible fashion and written in standard English?

PLOS ONE does not copyedit accepted manuscripts, so the language in submitted articles must be clear, correct, and unambiguous. Any typographical or grammatical errors should be corrected at revision, so please note any specific errors here.

Reviewer #1: Yes

Reviewer #2: Yes

**********

5. Review Comments to the Author

Please use the space provided to explain your answers to the questions above. You may also include additional comments for the author, including concerns about dual publication, research ethics, or publication ethics. (Please upload your review as an attachment if it exceeds 20,000 characters)

Reviewer #1: The manuscript entitled “ Spontaneous passage of common bile duct stones: predictive factors and impact on post-ERCP complications” aimed to identify clinical predictors of spontaneous passage and evaluate its association with post-ERCP complications. The authors concluded that spontaneous passage of CBDSs is an independent risk factor for PEP and younger age, smaller stone size, and a single stone are significant predictors of spontaneous passage.

The manuscript is well written, novel and the results may be of clinical relevance. However, there are some comments that need to be addressed.

- The diagnosis of CBDS was made using various imaging techniques. False-positive results are also possible. Please discuss the ‘false-positive rate’ for each technique and mention this in the text.

- The risk of PEP is influenced not only by patient-related factors but also, to a significant extent, by factors related to the examination. Factors such as cannulation of the pancreatic duct, contrast injection into the pancreatic duct and placement of a protective stent in the pancreatic duct were not mentioned. Nor was it mentioned whether rectal NSAIDs are administered for PEP prophylaxis. Please mention and discuss.

-What was the technical success rate of the ERCPs? How often was a pre-cut papillotomy necessary?

-Even a spontaneous stone passage can lead to pancreatitis. How was the distinction made between PEP and biliary pancreatitis caused by a spontaneous stone passage if this occurred shortly before the ERCP? If a clear distinction cannot be made, this should be noted under ‘possible limitations’.

- The mean duration from diagnosis of CBDS to procedure (ERCP/EUS) was 29 days. That is a quiet long time. This is surprising, given that a large proportion of patients presented with acute cholangitis (Table 1). National and international guidelines recommend early ERCP for acute cholangitis. This should be discussed, as should the limited applicability of these data to other patient groups who have easier access to prompt ERCP.

- Why was an EUS performed on some patients prior to a potential ERCP, despite their previously confirmed CBDS? Please explain.

Reviewer #2: In this manuscript Wanchaitanawong et al. report on the frequency and predictors of spontaneous passage of common bile duct stones in a retrospective cohort of 113 patients. The observed spontaneous passage in 28% of the patients and identify several factors associated with this outcome. Most importantly, they also observe a higher number of complications in patients with spontaneous passage underscoring the need to identify these before the procedure.

The study is technically sound and the manuscript is well written. The data are interesting.

However, a few points need to be considered:

- While the various predictors of spontaneous passage are certainly interesting, one very important aspect is inadequately covered: The predictive value of confirmation or exclusion of CBDS by appropriate imaging DIRECTLY before the ERCP. To this end, it is important to include this into the list of predictors. Roughly estimated, the p-value for positive EUS to exclude spontaneous CBDS passage should be 0.005 in this cohort and is therefore certainly relevant.

- This is also the most important and clinically relevant conclusion of the manuscript and should be made clearer in the discussion: In particular in cases with a substantial delay between diagnosis and intervention, EUS should be performed directly prior to ERCP to confirm or rule out CBDS.

- To identify cases, where the cumulative evidence from clinical predictors for spontaneous stone passage is so high that re-EUS might be omitted, I suggest the authors build a predictive model from these factors and indicate its sensitivity, specificity, NPV and PPV.

- The study design comes with the weakness that a subgroup of patients has previously undergone EUS. Thus, the passage group includes a subgroup that has not undergone ERCP and the non-passage group includes a subgroup, where non-passage has already been confirmed before ERCP. This introduces some - probably minor - inconsistencies. However, I suggest to provide (e.g. as Supplementary Data) a separate analysis only focussing on the ERCP cases without previous EUS.

- Please analyze whether the type of diagnostic mortality is associated with stone passage. Given the high number of diagnoses made with CT scan, I suspect that a high number of CBDS in this group were incidental findings on imaging performed for another indication.

**********

6. PLOS authors have the option to publish the peer review history of their article (what does this mean?). If published, this will include your full peer review and any attached files.

If you choose “no”, your identity will remain anonymous but your review may still be made public.

**Do you want your identity to be public for this peer review?**  For information about this choice, including consent withdrawal, please see our Privacy Policy.

Reviewer #1: No

Reviewer #2: No

**********

[NOTE: If reviewer comments were submitted as an attachment file, they will be attached to this email and accessible via the submission site. Please log into your account, locate the manuscript record, and check for the action link "View Attachments". If this link does not appear, there are no attachment files.]

To ensure your figures meet our technical requirements, please review our figure guidelines: https://journals.plos.org/plosone/s/figures

You may also use PLOS’s free figure tool, NAAS, to help you prepare publication quality figures: https://journals.plos.org/plosone/s/figures#loc-tools-for-figure-preparation.

NAAS will assess whether your figures meet our technical requirements by comparing each figure against our figure specifications.

# Author comment: Author response to Decision Letter 1

**DOI:** 10.1371/journal.pone.0351242.r002

5 May 2026

We have provided a detailed, point-by-point response to all comments raised by the academic editor and the reviewers in the attached 'Response to Reviewers' file. The manuscript and supporting information have been revised accordingly.

Attachment Submitted filename: Response to reviewers_4-5-2026.docx

# Peer review: Decision Letter 1

**DOI:** 10.1371/journal.pone.0351242.r003

**Authors:** Thomas Karlas

26 May 2026

Spontaneous passage of common bile duct stones: predictive factors and impact on post-ERCP complications

PONE-D-26-05028R1

Dear Dr. Thinrungroj,

We’re pleased to inform you that your manuscript has been judged scientifically suitable for publication and will be formally accepted for publication once it meets all outstanding technical requirements.

Within one week, you’ll receive an e-mail detailing the required amendments. When these have been addressed, you’ll receive a formal acceptance letter and your manuscript will be scheduled for publication.

An invoice will be generated when your article is formally accepted. Please note, if your institution has a publishing partnership with PLOS and your article meets the relevant criteria, all or part of your publication costs will be covered. Please make sure your user information is up-to-date by logging into Editorial Manager at Editorial Manager® and clicking the ‘Update My Information' link at the top of the page. For questions related to billing, please contact billing support.

If your institution or institutions have a press office, please notify them about your upcoming paper to help maximize its impact. If they’ll be preparing press materials, please inform our press team as soon as possible -- no later than 48 hours after receiving the formal acceptance. Your manuscript will remain under strict press embargo until 2 pm Eastern Time on the date of publication. For more information, please contact onepress@plos.org.

Kind regards,

Thomas Karlas

Academic Editor

PLOS One

Additional Editor Comments (optional):

-

Reviewers' comments:

Reviewer's Responses to Questions

**Comments to the Author**

1. If the authors have adequately addressed your comments raised in a previous round of review and you feel that this manuscript is now acceptable for publication, you may indicate that here to bypass the “Comments to the Author” section, enter your conflict of interest statement in the “Confidential to Editor” section, and submit your "Accept" recommendation.

Reviewer #1: All comments have been addressed

Reviewer #2: All comments have been addressed

**********

2. Is the manuscript technically sound, and do the data support the conclusions?

The manuscript must describe a technically sound piece of scientific research with data that supports the conclusions. Experiments must have been conducted rigorously, with appropriate controls, replication, and sample sizes. The conclusions must be drawn appropriately based on the data presented.

Reviewer #1: Yes

Reviewer #2: (No Response)

**********

3. Has the statistical analysis been performed appropriately and rigorously?

Reviewer #1: Yes

Reviewer #2: (No Response)

**********

4. Have the authors made all data underlying the findings in their manuscript fully available?

The PLOS Data policy requires authors to make all data underlying the findings described in their manuscript fully available without restriction, with rare exception (please refer to the Data Availability Statement in the manuscript PDF file). The data should be provided as part of the manuscript or its supporting information, or deposited to a public repository. For example, in addition to summary statistics, the data points behind means, medians and variance measures should be available. If there are restrictions on publicly sharing data—e.g. participant privacy or use of data from a third party—those must be specified.

Reviewer #1: Yes

Reviewer #2: (No Response)

**********

5. Is the manuscript presented in an intelligible fashion and written in standard English?

PLOS ONE does not copyedit accepted manuscripts, so the language in submitted articles must be clear, correct, and unambiguous. Any typographical or grammatical errors should be corrected at revision, so please note any specific errors here.

Reviewer #1: Yes

Reviewer #2: (No Response)

**********

6. Review Comments to the Author

Please use the space provided to explain your answers to the questions above. You may also include additional comments for the author, including concerns about dual publication, research ethics, or publication ethics. (Please upload your review as an attachment if it exceeds 20,000 characters)

Reviewer #1: (No Response)

Reviewer #2: (No Response)

**********

7. PLOS authors have the option to publish the peer review history of their article (what does this mean?). If published, this will include your full peer review and any attached files.

If you choose “no”, your identity will remain anonymous but your review may still be made public.

**Do you want your identity to be public for this peer review?**  For information about this choice, including consent withdrawal, please see our Privacy Policy.

Reviewer #1: No

Reviewer #2: No

**********

# Editor report: Acceptance letter

**DOI:** 10.1371/journal.pone.0351242.r004

**Authors:** Thomas Karlas

PONE-D-26-05028R1

PLOS One

Dear Dr. Thinrungroj,

I'm pleased to inform you that your manuscript has been deemed suitable for publication in PLOS One. Congratulations! Your manuscript is now being handed over to our production team.

At this stage, our production department will prepare your paper for publication. This includes ensuring the following:

* All references, tables, and figures are properly cited

* All relevant supporting information is included in the manuscript submission,

* There are no issues that prevent the paper from being properly typeset

You will receive further instructions from the production team, including instructions on how to review your proof when it is ready. Please keep in mind that we are working through a large volume of accepted articles, so please give us a few days to review your paper and let you know the next and final steps.

Lastly, if your institution or institutions have a press office, please let them know about your upcoming paper now to help maximize its impact. If they'll be preparing press materials, please inform our press team within the next 48 hours. Your manuscript will remain under strict press embargo until 2 pm Eastern Time on the date of publication. For more information, please contact onepress@plos.org.

You will receive an invoice from PLOS for your publication fee after your manuscript has reached the completed accept phase. If you receive an email requesting payment before acceptance or for any other service, this may be a phishing scheme. Learn how to identify phishing emails and protect your accounts at https://explore.plos.org/phishing.

If we can help with anything else, please email us at customercare@plos.org.

Thank you for submitting your work to PLOS ONE and supporting open access.

Kind regards,

PLOS ONE Editorial Office Staff

on behalf of

Prof. Thomas Karlas

Academic Editor

PLOS One
